# Exploring Eating and Nutritional Challenges for Children with Autism Spectrum Disorder: Parents’ and Special Educators’ Perceptions

**DOI:** 10.3390/nu12092530

**Published:** 2020-08-20

**Authors:** Noor Akmal Shareela Ismail, Nurul Syafinaz Ramli, Nur Hana Hamzaid, Nurul Izzaty Hassan

**Affiliations:** 1 Department of Biochemistry, Faculty of Medicine, Universiti Kebangsaan Malaysia, Kuala Lumpur 56000, Malaysia; 2 Centre of Rehabilitation and Special Needs, Faculty of Health Sciences, Universiti Kebangsaan Malaysia, Kuala Lumpur 50300, Malaysia; p94467@siswa.ukm.edu.my (N.S.R.); hanahamzaid@ukm.edu.my (N.H.H.); 3 Dietetic Program, Faculty of Health Sciences, Universiti Kebangsaan Malaysia, Kuala Lumpur 50300, Malaysia; 4 Department of Chemical Sciences, Faculty of Science & Technology, Universiti Kebangsaan Malaysia, Bandar Baru Bangi, Selangor 43600, Malaysia; drizz@ukm.edu.my

**Keywords:** ASD, nutrition, module, education, food behaviour

## Abstract

Autism spectrum disorder (ASD) is a complex neurodevelopmental disability that is frequently associated with food refusal, limited food repertoire and high-frequency single food intake mainly among children with ASD. Provision of nutrition can be very challenging due to the fact of these behavioural problems, either for the parents or special educators. Healthy nutrition is associated with providing and consuming nutritious food with results being in a good state of health. Semi-structured focus group discussions (FGDs) were conducted among 20 participants at a National Autism Centre to explore their understanding towards healthy nutrition. They were parents and special educators who were actively involved with children with ASD. A series of discussions were transcribed verbatim, and four researchers examined each transcript. Inductive analysis linking codes into main thematic categories was conducted using the constant comparison approach across the full data set. The outcome suggested that participants had limited knowledge relating to the proper dietary and nutritional needs of the children. The key messages from the discussion provide a foundation on the development of a nutrition education module which involves primary caretakers of children with ASD.

## 1. Introduction

Autism spectrum disorder (ASD) is a complex neurodevelopmental disability characterised by several diagnostic criteria such as deficits in social communication and social interaction and restricted, repetitive patterns of behaviour, interests or activities [1]. In 2012, the combined estimated prevalence of ASD among the Autism and Developmental Disabilities Monitoring (ADDM) Network sites was one in 69 children aged eight years [2]. The estimated prevalence was significantly higher among boys aged eight years (23.4 per 1000) than among girls with the same age (5.2 per 1000) [2].

Due to the symptoms of ASD, these children are frequently associated with disruptive mealtime behaviour, such as severe food refusal, limited food repertoire and high-frequency single food intake [3], which are closely related to sensory processing difficulties (i.e., texture, smell and taste) [4]. This is plausibly due to the rituals [5], repetitive behaviours [6], obsessions [7] and refusal of foods according to sensory issues [7,8]. As a consequence, children with ASD are mostly exposed to the risk of low nutritional status. Ranjan et al. (2015) [9] reported that the prevalence of overweight among children with ASD and typically developing (TD) children is 19% compared with 16% while obesity is 30.4% compared with 23.6%. Children with ASD, in general, are also reported to have lower serum levels for the reference ranges for pantothenic acid, biotin, folate, vitamin B12, vitamin D and vitamin E [9]. More specifically, children with ASD generally eat too many calories from either protein, carbohydrates or fat (or the combination of these three nutrients), and they typically do not consume enough of vitamins A, E and D, folic acid and calcium [10,11].

The Malaysian Dietary Guidelines (MDGs) encompass 15 key messages and 67 recommendations for healthy children and adolescents from birth to 18 years of age; they cover the whole range of food and nutrition issues, from the importance of consuming a variety of foods to guidance on specific food groups and messages encouraging physical activities, consuming safe food and beverages and making effective use of nutrition information on food labels [12]. However, there are no special dietary guidelines specially curated for children with ASD in Malaysia at this point in time.

Children with ASD are mainly taken care of by parents or a caregiver who mostly contribute to the decision-making process of food provisions for their child. With limited research conducted on assessing the nutritional knowledge level among primary caretakers of children with ASD, findings have reported a lack of knowledge relating to nutrition [9,13,14,15]. However, some showed positive perceptions towards specific special diets (restrictive diets) regardless of the low level of understanding of the diet [15]. Therefore, this study aimed to explore the nutritional knowledge among parents and special educators of children with ASD and how it reflects on their food provision practice through a qualitative approach.

## 2. Materials and Methods

### 2.1. Research Design, Sample and Data Collection

Open-ended, semi-structured focus group interviews were conducted among parents and special educators of children with ASD in this qualitative cross-sectional study. Participants were recruited using a convenient sampling method from the national (Malaysia) autism centre. This early intervention centre is government funded for children with autism and their families. The centre provides services related to early intervention therapies and education in preparing students for mainstream schools. Intervention strategies implemented in this centre incorporate evidence-based practice and naturalistic teaching with the cooperation of transdisciplinary groups and the active participation of parents. This centre was chosen as it is the national reference centre for Malaysia that gathers students from the whole country with similar socioeconomic status. All students who attended this centre were also given similar exposure in all areas. However, the curriculum has no specific input related to basic nutrition input neither to the students nor their parents. Therefore, this centre was found to be a suitable setting for achieving the study’s objectives.

Participants were allocated into separate groups of parents and special educators to ensure similar background when answering the interview questions. Parents were either the mother or father of students attending the national centre for autism of Malaysia situated in Kuala Lumpur, for at least six months, whereas special educators were those with a minimum of one-year experience. Students of the centre were ASD cases from a selected socioeconomic status, referred mainly from a paediatric specialist of hospitals around Malaysia. Selected candidates were then enrolled for half-a-day three days a week class. The special educators were those with occupational therapy, speech therapy and physiotherapy backgrounds and none with nutrition or dietetics degree. Approval for this study was granted by the university ethics review board (UKM PPI/111/8/JEP-2018-665). Basic descriptive demographic details were collected from the participants including the child’s age, gender, years diagnosed with ASD, household income and education level of parents and special educators.

The focus group discussions were conducted and recorded by a moderator (one nominated researcher, NSR) who underwent training by an experienced qualitative researcher. There were eight series of interviews conducted for the parents and four series for special educators. The open-ended interview questions (Table 1) were focused on several topics: knowledge and awareness on nutritional issues, understanding of nutritional requirements for children with ASD, limiting factors that inhibit them from practising the best dietary intake for their children and their concern about their child’s health. Participants were also allowed to add relevant information at the end of the interview. The moderator also took notes in addition to the recordings.

### 2.2. Data Analysis

The recorded sessions were transcribed verbatim into word documents. These data were then reviewed against the digital recording to confirm accuracy. The coding process was conducted by two of the researchers (N.S.R. and N.H.H.). Data were subjected to thematic analysis [16] and analysed by the research group (N.S.R., N.H.H., N.I.H., N.A.S.I.) individually for emerging themes and subsequently for main themes in several group discussions until a consensus was reached. Final themes and subthemes were then agreed on, with relevant and substantive quotations were selected as exemplars.

## 3. Results

### 3.1. Participants Description

All parents had a child diagnosed with ASD for at least six months, and all of them attended a special class at the National Autism Centre, Kuala Lumpur. Table 2 reveals that more females participated in this study (57%) compared to males (43%). The age of all participants was in the range of 28 to 43 years old. The highest education was a Master’s degree, and the lowest was primary education. Most of them were working as public workers (71%) and self-employed (29%). There was a mixture of household incomes, and most of the parents acquired less than RM5000 a month, which represents low- and middle-income groups (100%). The age of their children ranged from 4 to 7 years old. Only seven of them had a healthy weight (50%), and the remaining were underweight (43%) or overweight (7%). For the special educators (*n* = 6), they had graduated with degrees with various backgrounds in allied health science but not in nutrition and/or dietetics.

### 3.2. Themes

**Figure 1:**
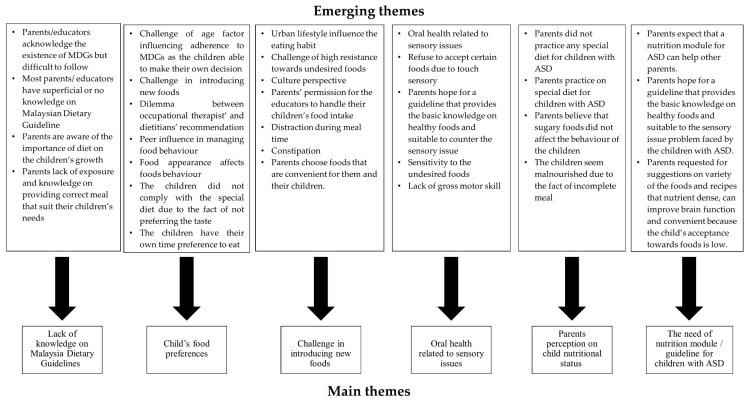
The summary of emerging themes based on the focus group discussions.

The focus group discussions yielded 30 emerging themes that highlighted the nutritional issues associated with children with ASD based on the semi-structured questions (Figure 1). We further collated the themes and summarised them into five overarching themes highlighted from the discussion (Table 3). We labelled parents as “P” and Special Educator as “E” in our transcripts. The “I” indicates the *n*th interview session for all the discussion.

#### 3.2.1. Theme 1: Lack of Knowledge and Adherence to Malaysia Dietary Guidelines

The discussion started when both parents and special educators were asked about the knowledge of the Malaysia Dietary Guidelines (MDGs). When further prompted on the MDGs, the respondents managed only to pronounce a superficial description of the guidelines. They also mentioned that it was imperative to adhere to essential healthy eating by consuming vegetables and fruits, avoiding junk foods and having balanced meals without describing the actual vital messages of the guidelines.

“The guidelines tell us to practice healthy eating. So, it should be good for children with autism as well.”.

(P1I4)

Another remark by the parent, “It [MDG] talks about healthy eating, eating vegetables, fruits, carbohydrates, protein and milk, right? So basically, it suits these autism children.”

(P1I3)

Five out of fourteen parents also discussed several challenges and limiting factors that they faced in practising the guidelines for their children.

“The guidelines are handy if we follow it by the book, but we usually make do with everything that we have at home.”

(P1I1)

“Yes, it is hard to follow [the guidelines] because my kid is a picky eater.”

(P1I2)

Another parent said:

“We are aware of the healthy nutrition from the guidelines, but we are dealing with autism children, like my kid he is very picky because he has sensory issues.”

(P1I4)

A special educator has also added that, “[The guidelines] have advised the children to avoid junk food and replace it with fruits; in reality, the children hate to eat fruits.”

(E2I1)

However, there was one parent who highlighted the importance of promoting the guidelines on TV and radio, so the parents know the existence of the MDGs.

“But for me, in Malaysia, this seems to be less about promoting the [Malaysia] nutrition guidelines for children. We can see more junk foods and fast food ads than the ads in encouraging healthy eating. Right? I do not think parents know that the guidelines exist.”

(P1I5)

#### 3.2.2. Theme 2: The Challenge of the Child’s Age Factor Influencing Adherence to Malaysian Dietary Guidelines

Some parents and special educators reported that when their children reached an individual developmental phase or age, the parents will attempt to introduce new food into their children’s diet. Nevertheless, it is not a straightforward approach to ensure that children with ASD can accept the food. These participants agreed that children’s age factor might become a challenge for them to adhere to the Malaysian Dietary Guidelines. Findings showed that their children were more acceptable to any food introduced before reaching the toddler phase. However, as they grew older and to the toddler age, they seemed to have power in deciding the type of foods that they preferred, mostly based on the appearance and texture of the foods.

A parent said, “Growing up, he can dictate his preferences, so he will reject all the foods that have been introduced to him.”

(P1I1)

The same parent also added, “When he can eat on his own, he becomes picky and only eats food that he likes. I tried to be there and introduce new foods, but I am afraid it can interfere with his behavioural therapy.”

(P1I1)

Nevertheless, the same parent also without fail, always introduces new healthy food to her child, to make a variety of daily menu. A special educator shared that there was a student who only loves a specific brand (of food). Since he can speak well, he knew where to get his favourite food. She gave an example,

“If he likes a bowl of chicken rice from shop A, he only eats from shop A, not B or C. He knows how to differentiate the taste.”

(E1I3)

#### 3.2.3. Theme 3: Challenges in Introducing a New Food

It is worth noting that parents and special educators are facing different challenges in introducing new food to children with ASD, which may be due to the fact of food appearance, peer influence and urban lifestyles. Food appearance is heavily dependent on the colour and texture.

One parent said, “Apparently, I just found out that kids with autism love soft and pastel colours. That is why even if I wanted to give him the gravy (with rice), he will contemplate on it because he did not want the food to have striking colour and be gooey. For that, I always use vegetable broth for cooking rice (to give a subtle colour).”

(P1.1)

However, a special educator said, “The children are very selective with the food appearance. Whenever parents provide packed food, such as rice with small, diced carrots, the students will notice and refuse to eat.”

(E1I2)

“There was a parent who always gives her kid milk and cereal. I told the parents ‘do not give him [a bottle, or else he would not drink’. I suggested to introduce rice [instead of milk], but he only ate a little and then threw up.”

(E2I1)

Food appearance also related to sensory issues. Nine parents experienced this same problem.

“My child does not know how to chew properly. He prefers porridge than rice. If the porridge contains shredded chicken, he will notice it and try to spit it out.”.

(P1I3)

Another parent shared the same notion, “He has a little sensory problem. Up to this day, he likes to put food in his mouth first, if he does not like it, he will spit it out. Usually, he likes to spit out hard [texture] food. He may not be able to chew; that is why he always loves soft food.”

(P4I4)

The smell of the food also contributes to food preferences. One parent said that his child was susceptible to smell. “Some people cannot smell it. My kid can smell the chicken, and he does not like it. Moreover, he keeps on making gagging noise.”

(P2I1)

Almost half of the participants (parents and especially special educators) mentioned that peer influence could help in introducing a new food, but this must be practised for a few sessions before it takes effect.

“If a friend at school eats many vegetables, he would follow, and at home, he would eat a lot.”

(P1.1)

“Usually the students will try to eat their friends’ food because they are not familiar with the ones provided by their parents.”

(E1.1)

Lack of motor skills during eating can be a challenge by both parents and special educators. Most of the special educators shared the same view on the inability of children with ASD to sit and independently put the food to their mouth, and they always require extra help. The special educators said the children need to be assisted during lunch; otherwise, they will not be eating.

“Sometimes, he does not want to eat (on his own), even though his mother provides the food that he likes. He plays, then he will get distracted to find his toys. So, I always assist him to eat.”

(E2I1)

“Yes, but that one depends on the consent of the parents if the parents say they want their child to be assisted by us, so the child must learn (how) to eat the food. So, if the child refuses, we have to assist him to eat properly.”

(E1I2)

Living in the city also can influence the food choices made by both parents and children with ASD. Most of the parents opted for processed food, preferably more than homemade food because they are working parents, and it is easier to buy rather than to prepare the food. They think that if the food is (deemed) healthy, it can be bought to save their time in managing the behaviour of their children. They tend to choose the same type of food in their daily lives which mainly consists of high calories and lower nutrient-dense foods.

“We do not focus on the food because we want him to focus on his behaviour. We buy what is available. I always buy food that everyone will eat.”

(P2I7)

Two special educators agreed that the phenomenon is inevitable since most of the parents are working and they are comfortable buying packed food as luncheon to school.

“It is like that [buying processed food than homemade] because we live in the city….”

(both E2I1 and E1I2)

“Most parents are working, they are busy, so usually they buy food on the go. Examples are food in packets; like cookies, nuggets and things that are easy to prepare, fried food.”

(E1I4)

#### 3.2.4. Theme 4: Oral Health Related to Sensory Issues

Three out of fourteen parents have reported that their children bear oral health issues exemplified by dental associated damage and inability to chew caused by feeding behaviour. They further iterated this was due to the fact of their children’s habit and preferences towards hard and crunchy textured foods.

“Because he eats so much of crunchy food, which is why his (molar) teeth chipped a little. That is why the dentist suggested crowning as a treatment, due to lack of self-control from eating that [crunchy food].”

(P1.1)

Upon prompting, a special educator also mentioned that, “Most of children with ASD have broken tooth due to the habits of eating sweets and crunchy foods.”

(E1I2)

#### 3.2.5. Theme 5: Parents Perception of Child Nutritional Status.

As commonly reported, the children with ASD are usually associated with low nutritional status [9,17,18], and this was not an exception in this focus group discussions. Several parents reported that due to the mealtime behaviours, such as food refusal and food selectivity, their children seemed to look malnourished, either being underweight or overweight, that was mainly caused by long-term consumption of unbalanced nutritious meals.

“If it is for the kids in general, he has enough nutrition. Vegetables, chicken, fish that is it. And fruit. I like to cook vegetable fish soup. So, for the kids in general [I think] he has enough nutrition.”

(P1I5)

Another comment was, “Basically, [he can eat] vegetables, fruit, milk. I know those snacks are not good. However, that is what he always wants to eat.”

(P1I7)

Special educators also thought that parents lacked exposure and knowledge on providing nutritious meals for their children. This was reported based on the observations made during lunchtime when the children with ASD mostly drank milk or milk mixed with blended cereal to accommodate sensory issues, which are thought to provide energy to the children. Some of the parents knew about special diets, especially gluten-free and casein-free diets, and tried to implement them in their children’s diet.

“My wife has read about gluten-free, casein-free. Autism children cannot eat bread and milk, right? Sometimes when we found gluten-free biscuits at the bigger mall, we tried to buy it, but he does not seem to like it that much. So, I think it [the special diet] does not work.”

(P4I4)

“For example, parents know about like gluten-free, sugar-free food. They keep reminding us [the teachers] not to give any other food that has not been provided by the parents.”

(E1I1)

#### 3.2.6. Theme 6: The Need to Have a Nutrition Module or Guidelines for Children with ASD

All participants, especially parents that had been interviewed in this study, were generally worried about their children’s nutritional status. This wariness was due to the fact of their children’s picky behaviour during mealtime and how it would affect their weight. They were very expectant towards specific guidelines that would provide basic knowledge on healthy food and would be suitable to the sensory issue problem faced especially by children with ASD.

“Maybe there is a need for a guideline specifically made for parents with children with ASD on to know how to control diet and how to eat properly. Examples of such snacks should be reduced, with added high-quality protein, healthy food. Better not to give them [children with ASD] calorie dense food like cake and chocolate.”

(P3I1)

Parents also requested for suggestions on a variety of foods and recipes, because the child’s acceptance towards foods was low.

“Because my child is a picky eater, maybe you can suggest many ways to get him to eat. Because I can see other children are forced to eat by their parents. Otherwise, they do not want to eat.”

(P1I6)

The parents also wished for suggestions on menus or recipes that are easy to prepare for working parents and packed with nutrients at the same time.

“We are mostly working parents, and we want an easy recipe to cook for our (autism) children. A meal that is easy and nutritious.”

(P1I5)

Some of the parents and special educators wished to discover more food that is good for the brain and improves behaviour.

“Perhaps, if we can know a certain type of food that very nutritious and can be eaten by picky eaters.”

(P1I6)

“If there is a way that he can control his behaviour through diet, and what mechanism that can change it?”

(E2I1)

## 4. Discussion

A quantitative study that was conducted prior to the current qualitative study has found that parents with children with ASD have inadequate awareness and knowledge of nutritional issues of their children (unpublished data) [19]. Earlier, our previous preliminary study revealed that inadequate knowledge was significantly correlated with low nutritional status of their children (*p* < 0.05) [19]. The data are hence calling for the development of a nutrition module to educate parents and special educators in implementing the proper nutritional and dietary intake for children with ASD. Having nutrition knowledge among parents or caregivers is essential in determining children’s diet, which is consistent with the few existing studies on the effects of nutrition awareness and knowledge of the parents on the children’s diet. Reports on surveys of nutritional knowledge of both parents and teachers, such as in the US [20], Greece [21], Australia [22] and Malaysia [23], demonstrated the importance of nutrition knowledge among parents on their children’s diet. For this, we need to identify and discuss the themes extracted from our qualitative study.

### 4.1. Theme 1: Lack of Knowledge and Adherence to Malaysia Dietary Guidelines

The revised Malaysian Dietary Guidelines (MDGs) are a product of evidence based on both nutrition and physical activity recommended by experts in the Nutrition Society of Malaysia and were carefully compiled as nutrition messages for the public. It emphasises the promotion and usage through various platforms including educational activities for children [24]. Among the real challenges in ensuring and convincing the public is to adopt these dietary guidelines to solve some of their nutritional problems. The understanding of five key messages in the MDGs evaluated among adults living in Kuala Lumpur [25] revealed a moderate understanding, as they are unclear with some keywords such as serving size and sedentary habits. This observation coincides with our preliminary unpublished quantitative data relating a lack of knowledge among the parents and caregivers [19]. The plausible reason is due to the lack of awareness given by the government and non-governmental bodies in educating parents and caregivers with children with ASD. In addition, it magnifies the role of nutrition education [26] in promoting healthy eating patterns among adults.

It is also imperative to simplify the nutrition messages to enhance their understanding and ensure the public are exposed to recent and accurate dietary recommendations. Moreover, the MDGs need to be promoted by utilising different media formats and channels to gain appropriate public knowledge on healthy eating and active living. Additionally, a significant correlation associated with the level of understanding of the MDGs with education level and occupational status coincided with our demographic data. Low-income household populations perceived healthy food as a secondary necessity and, hence, less prioritised. Thus, they are inclined to purchasing food that is high in fat and/or calories, as it is cheaper than perishable wholesome food. Nutritional knowledge has also been reported to be independently related to poor diet quality with low compliance to the Malaysian dietary recommended intake. A constant effort to improve diet quality among low educational qualifications and lower-income households is crucial to prevent diet-related non-communicable diseases [27].

### 4.2. Theme 2: The Challenge of Child’s Age Factor Influencing Adherence to Malaysian Dietary Guidelines

Nutritional patterns that are formed during childhood may contribute to the prevalence of malnourishment among school children in Malaysia [28]. Primary school children are vulnerable to being at risk of poor dietary behaviours [29], where they are found to consume unhealthy snacks over fruits and vegetable. This may lead to lower cognitive performance at school [30]. Our participants expressed that children’s age factor may become a challenge for them to adhere to the Malaysian Dietary Guidelines. A comparable cross-sectional study observed poor adherence of Swiss children aged 6–12 years old with the dietary guidelines. Younger children meet the fruits and cereals requirement by which older children are more likely to display better adherence for egg intake [31]. Other studies established that the increasing soda intakes are a trend with older age [32] due to the presence of extrinsic factors such as peer group influences, lower parental control and autonomy in the choice of food [33].

In contrast, another study demonstrated a non-linear association between age and food preference [34]. For children with low-income family subjects, intrinsic factors such as social factors dictated their choice of food reference regardless of the age factor. These parents are more anticipated in assuring the independence of their children in society. Moreover, the community may occasionally pamper them with their food reference as an indication of welcoming and encouraging them to fit into society. A combination of the behavioural and nutritional invention has proven to increase food acceptance and consumption for a child with ASD [35]. Local governments can also play a role in advocating both parents and children to consume healthy, safe and affordable foods, while at the same time making efforts in limiting access to calorie-dense, nutrient-poor foods [36].

### 4.3. Theme 3: Challenges in Introducing New Food

Children with ASD are more prevalent in choosing processed foods over fruits and vegetables [9]. They are also very particular towards refusing food based on its texture especially in slippery, and soft foods [37]. Additionally, some children with ASD may only eat food that can be either sweet, salty, sour or bitter [38]. Children with ASD also tend to prefer a particular texture over another such as crunchy or smooth or they prefer to have the same food [39] at a consistent temperature [38]. Our study found that food appearance was mostly discussed among the parents in introducing new food to their children. It concurs with a study that revealed the most mothers of children with ASD identified this as one of the feeding challenges [40]. Early identification of feeding challenges in ASD is necessary to minimise adverse health outcomes and strained parent–child relationships [41]. Difficulty with food textures due to the sensory problems thus affect food selectivity [42]. Food selectivity is the major problem among children with ASD, and this is shown through food refusal, eating rapidly, difficulty in chewing, stealing food from other friends and vomiting [7], and these occurrences were similarly found in our semi-qualitative study. Children with ASD also face difficulties in accepting new food which is commonly associated with behaviours problem. This difficulty is affected by sensory processes, perception of taste, irrational fear of strange things, environmental factors, parental and peer modelling and feeding practices [43].

Parents often struggle, especially in school’s homework, managing to eat, doing chores and intimate conversations during mealtime [44]. Many children with ASD face problems focusing on a task given, and this can be seen in that they often are in distress to stay put while eating [9]. To date, there are three different structured programs to introduce new food through food shaping [45], reinforcement [46] and nutrition information [47]. If the child is sensitive to textures and smells, new foods can be slowly introduced by educating them while doing groceries, especially in learning about new foods, subsequently preparing meals together. Exploring new foods through food play and peer influence can also be incorporated in the plan of introducing new type of foods. Such approaches are similarly found in our finding, where experience at school with friends does help in getting them to eat in a household environment. This experience allows children with ASD to be familiar with the new foods [48,49]. Repeated exposure is also essential to allow the child to have a sense of control. Another approach is to engage with the child by smelling, licking or tasting the food [36]. There is an emerging approach related to this area which is called food chaining. Food chaining is another way to offer the child new experience in expanding the variety of foods along the process. This method is the most sought-after approach for a picky eater to enable them to attempt to eat new foods that are similar to the one they currently love.

### 4.4. Theme 4: Oral Health Related to Sensory Issues

Oral health is significant in determining the dietary intake of these children, as a dental health issue will limit the variety of foods taken. Sensory sensitivities during feeding time is usually associated with anxiety that can lead to food avoidance [50]. It will subsequently cause behavioural problems in children with ASD who are unable to describe their distress [51]. Sensory experiences can be grouped into two categories—hypersensitive and hyposensitive—which can affect oral health among children with ASD. Therefore, adequate tooth brushing is often reduced in children with ASD [52]. Our participants also expressed their concern for their child’s oral health, and this accords with few studies that found dental and oral care is very problematic for children with ASD [52,53]. Most autistic children are seen to either have poor or fair oral hygiene when compared to healthy control subjects [54,55]. They also had significantly exhibited various oral problems; thus, they needed more restorative dental treatment as compared to normal children [56].

### 4.5. Theme 5: Parents’ Perception of Child Nutritional Status

The importance of a sound nutritional status among children is paramount in assuring healthy development of a physical and cognitive function. As children have not reached the level of maturity to assess their nutritional status, parents play an essential role in ensuring sufficient nutrition is provided to their children. Parental perception of nutritional status, particularly diet quality, is influenced by many different social, biological economic and psychological factors [56]. Parents who can identify and show concern of their child’s unhealthy weight have been associated with higher readiness in improving the family’s diet quality and effectively manage the child’s eating and exercise behaviours [57]. This can be improved by implementing restrictive diet such as casein-free and/or gluten free diet as dietary intervention [58,59]. Our study found that parents who had a higher educational background inquired more about their children’s nutritional status. Others thought that the nutritional status was sufficient and similar to a typically developing child, so they did not question further and seek extra information. By communicating with the many experts, including nutritionist, parents may know what is lacking in their children’s diet and discussion is one of the ways on how to improve it. The experts were always updated with recent development pertaining to the new findings in nutrition among children with autism. Based on the latest studies, there are associations between micronutrient deficiencies with neurodevelopmental disorders, and low nutritional status of pregnant mothers can be a risk for such disorders including ASD [60]. Recently, there was a study linking low iodine levels in the first trimester of pregnancy and processing disorders of the child including children with ASD [61]. Additional deficiencies in iron [62], magnesium [63] and vitamins A and D [64] are often seen to exacerbate symptoms in children with ASD. It is therefore worth noting that speaking with experts may benefit parents to create more awareness.

Children with ASD are mostly taken care of by their parents including the provision of food for the child. Therefore, parents’ perception is very crucial among families with children with ASD. Parents from the current study were found to have limited nutrition knowledge similar to other studies [9,65,66]. Nevertheless, several parents also showed proper nutrition knowledge in the effort to improve their children’s diet despite reporting shortfalls in the implementation. This might be due to the fact of several restrictions such as food refusal by their children and limited communication with health professionals. The feedback given by the parents was a lack of awareness from the government and non-government organisation. However, these agencies are continually making efforts in updating modern techniques and interventions for children with ASD. The underprivileged families also often missed events conducted by these agencies due to the fact of both intrinsic and extrinsic factors. Nevertheless, a study on parents of children with ASD in Malaysia was found to have high adaptability levels and had the strength and determination in improving the underprivileged conditions [67]. More proactive efforts by healthcare professionals, including nutritionist were found to be crucial in disseminating information to both parents and special educators. Therefore, with proper education materials, parents’ nutrition knowledge can be improved, which later increases the awareness level to initiate actions towards improving the nutritional status of their child.

### 4.6. Theme 6: The Needs to Have a Nutrition Module or Guidelines for Children with ASD

The main calling for the development of a nutrition module specially catered to children with ASD is because they exhibit a higher prevalence of overweight and obesity as compared to the typical children. It is crucial to have a well-developed nutrition module in assisting parents to provide the best nutrition for their child. With the rise of diagnosis and awareness among the community and abundance of information available online, particularly from unreliable sources, it is timely to have a proper module to avoid malpractice that may be detrimental to the children. A well-thought module can also be a medium to assist stress management that is closely related to this population, mainly associated to social support, the severity of autism symptoms, financial difficulty and parents’ perception and understanding toward ASD [46]. Many parents opted for the non-evidence-based information as guidance which they perceived as the best nutrition option for their children.

With other ASD characteristics involving deficits in social communication and social interaction, managing food intake among this group is more challenging than typical children. This study showed that the majority of parents of children with ASD agreed that having a nutrition module would benefit them mainly in assisting in meal planning and food provision for their child. Several key factors need to be considered in developing nutrition modules such as a visually based, interactive format and the need to be culturally appropriate [68]. This is to ensure that effective communication can be delivered as a crucial first step in advancing one’s understanding [69] towards nutritional knowledge.

### 4.7. Limitation of the Study

Having the privilege to conduct this study in a national centre brings many advantages. Nonetheless it has its own limitation, because as a national early intervention centre for children with ASD, the number of students recruited in this centre was limited in order to keep a small ratio of special educators to students which was reflected in the small sample of this study. Although this sample size can provide some interesting pilot data, it has to be cautiously used as a general conclusion. The findings can also be strengthened with additional clinical data.

## 5. Conclusions

A reflective approach was one of the pivotal steps in delivering updated information related to healthcare practice aiming for parents and special educators, especially in the ASD population. With the wide range of ASD spectrum, a thorough approach with multidisciplinary efforts must be made in improving and enhancing the knowledge. The information can then be executed through management process optimisation. This study mainly identified essential gaps in nutritional knowledge among parents and special educators for children with ASD. Therefore, it is time to develop an educational tool, such as a comprehensive nutrition module specialised for the Malaysian context, aiming to help parents and special educators in implementing the best approach in achieving a healthy lifestyle for their children with ASD.

## Figures and Tables

**Table 1:** Interview guide for focus group participants.

	Interview and Probe Questions for Focus Group Discussion
1.	**How long does it take for your child to get an education at the National Autism Centre?** Type of class? Any changes in the behaviour? Food preferences? Nutrition?
* 2.	**How well do you know about healthy eating based on the Malaysian Dietary Guidelines (MDGs)?** What do you think about the appropriateness of this nutrition guide for children with ASD?
* 3.	**Share the problems you are having with managing your child’s/students’ nutrition?** What are the problems you face in managing children with autism? Any problems in terms of communication? Any problems in terms of behaviour? Any problems in terms of cognitive and motor skills?
* 4.	**What do you think of your child’s/students’ diet and physical growth patterns?** pIs it similar as compared to his/her peers or siblings at the same age?
* 5.	**Can you share your experiences on nutrition specifically for children with autism?** Give an example. How much do you believe that the diet is effective? Will you engage in this practice with your children? What are your reading resources? Why do you believe that nutrition is not necessary for children with ASD?
* 6.	**If you are given an opportunity, what information would you like to know from a nutritionist/dietitian on the nutrition of children with autism?** How to manage picky eaters? Constipation/digestive problems? Any other frequent illnesses? What are the best dietary practices? Mealtime? Type of food? Type of diet? Acceptance of children?

* Questions asked to both parents and special educators.

**Table 2:** Participants’ demographic data.

Parents (*n* = 14)			Special Educators (*n* = 6)
Gender	Female	8	5
	Male	6	1
Mean Age	Female	36.3	31.2
	Male	33.0	30.0
Education level	Advanced Degree (Master, PhD)	1	0
	Tertiary (Degree)	8	6
	Secondary (High School Diploma)	4	0
	Primary	1	0
Occupations	Not working	0	0
	Public officers	10	6
	Freelancer	4	0
Household income	>RM 5000	0	N/A
	RM 3001–RM 4999	4	N/A
	<RM 3000	10	N/A
Child’s Age	4–5 years old	7	N/A
	6–7 years old	8	N/A
Child’s BMI	Underweight	6	N/A
	Normal	7	N/A
	Overweight	1	N/A

**Table 3:** Quotes from different FGD sessions to highlight the main themes.

Main Theme	Quote
Lack of knowledge and adherence to Malaysia Dietary Guidelines	“We know we have to provide healthy diets to these kids, but autism kids tend to be picky eaters. Even ordinary children are picky lest to mention autism children. My kid has a sensory problem. So, it is a little hard to follow.” (P4I4) “It promotes healthy eating. I understand that a complete meal must consist of vegetables, side dishes and rice.” (E3I2)
Child’s food preferences	“Because he cannot eat his favourite food, when we introduced new food, he can be on hunger strike until we give him what he wants.” (P2I2) “Yes, we need to avoid junk food and eat more fruits, but they refuse to eat that.” (E2I1)
Challenge in introducing new foods	“When I tried to tell him to eat the rice, he will always follow, but if I introduced carrot in his rice, he could always see that, and he will remove them.” (P3I3) “Since he refuses to try another food, he can go on a hunger strike, and he can starve until we feed him food that he wants.” (E1I2)
Oral health related to sensory issues	“Because he eats so much, that is why his tooth chipped. The doctor advised for crowns treatment because he cannot control himself from eating that (hard texture food).” (P1I1) “I noticed some of the students have crooked teeth.” (E3I1)
Parents perception of child nutritional status The needs to have a nutrition module/guideline for children with ASD	“I always think that my kid’s diet is not sufficient. It seems that her weight growth is not the same as other kids. The other kid is a little fat, and she is underweight.” (P2I4) “We would like to see a good nutrition module, at least there is a guideline for us. It will be an alternative for parents to better understand their nutrition. What we can and cannot do.” (P2I1) “If there is a way (a guideline) that he can control his behaviour through diet, and what mechanism that can change it?” (E2I1)
